# Factors affecting the outcomes of tirofiban after endovascular treatment in acute ischemic stroke: Experience from a single center

**DOI:** 10.1111/cns.14058

**Published:** 2023-01-04

**Authors:** Yuan Wang, Hongrui Ma, Qihan Zhang, Feiyang Jin, Yi Xu, Qingfeng Ma, Xunming Ji

**Affiliations:** ^1^ Department of Neurology, Xuanwu Hospital Capital Medical University Beijing China; ^2^ Department of Neurosurgery, Xuanwu Hospital Capital Medical University Beijing China

**Keywords:** acute ischemic stroke, endovascular treatment, factors, GP‐IIb/IIIa inhibitor, tirofiban

## Abstract

**Aims:**

To investigate the predicted factors influencing the outcomes in acute ischemic stroke (AIS) patients who received tirofiban after endovascular treatment (EVT) and the optimal administration of tirofiban.

**Methods:**

In this retrospective study, AIS patients who received EVT followed by tirofiban between January 2017 and October 2021 were enrolled. The dose and duration of tirofiban were adjusted by trained clinicians according to the patient's clinical status. A reduction of at least four points on the National Institutes of Health Stroke Scale (NIHSS) after tirofiban compared with that before tirofiban was defined as an effective response. A modified ranking scale (mRS) of 0–2 was defined as a favorable outcome at a 90‐day follow‐up.

**Results:**

A total of 260 consecutive patients were enrolled, and 36.5% of patients achieved a favorable outcome. The modified thrombolysis in cerebral infarction (mTICI) 2b‐3 occurred in 93.5% of patients. Symptomatic intracerebral hemorrhage (sICH) occurred in 6.2% of patients, and the mortality at 90‐day follow‐up was 16.9%. Duration of tirofiban >24 h (adjusted OR: 2.545; 95% CI: 1.008–6.423; *p* = 0.048) and effective response to tirofiban (adjusted OR: 25.562; 95% CI: 9.794–66.715; *p* < 0.001) were related to the favorable outcome (mRS 0–2). Higher NIHSS (adjusted OR: 0.855; 95% CI: 0.809–0.904; *p* < 0.001) and glucose level on admission (adjusted OR: 0.843; 95% CI: 0.731–0.971; *p* = 0.018) were predictive for the unfavorable outcome (mRS 3–6).

**Conclusions:**

An effective response to tirofiban is an independent factor in predicting the long‐term efficacy outcome, and extending the duration of tirofiban is beneficial for neurological improvement.

## Graphical abstract

An effective response to tirofiban is an independent predictor for neurological function independence at 90 days. Increased admission NIHSS and glucose level may adversely affect the outcome of EVT combined with tirofiban. The optimal duration of tirofiban treatment to achieve a good neurological outcome was 36 h.

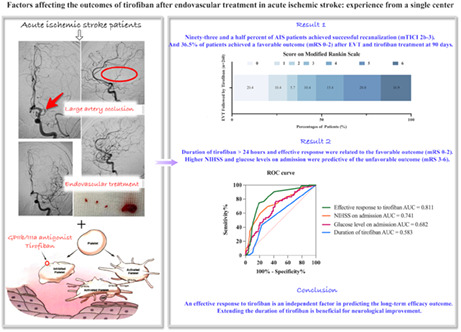

## 1 INTRODUCTION

Ischemic stroke has become the second leading cause of death over the last three decades, resulting in high mortality and morbidity worldwide.^1^ Intravenous recombination tissue plasminogen activator (rt‐PA) has been widely recommended as a standard treatment for acute ischemic stroke (AIS) in most clinical practice guidelines.^2^ However, only about 5%–20% of AIS patients received rt‐PA treatment, which is restricted by the narrow therapeutic time window, and only 2% of stroke patients could be beneficial from this treatment regimen.^3^, ^4^ Endovascular intervention can restore blood flow promptly, and thrombectomy with stent retrievers is now recommended as the standard care for AIS with a proximal large vessel occlusion (LVO).^5^, ^6^ However, as inherent damage to the endothelial cells lining of a blood vessel, endovascular treatment (EVT), such as mechanical thrombectomy (MT), angioplasty, and stenting, lead to local platelet aggregation, contributing to subsequent early reocclusion and thromboembolic complications.^7^ Numerous patients undergoing vessel recanalization following EVT were susceptible to acute reocclusion or late stenosis during subacute to chronic stages.^8^

Tirofiban, a glycoprotein‐IIb/IIIa (GP IIb/IIIa) inhibitor, which inhibits the final pathway of platelet aggregation and inhibits thrombosis in a more rapid approach,^9^ has been wildly used in patients with the acute coronary syndrome.^10^, ^11^ The effect of platelet aggregation blockade of tirofiban is dose‐dependent and can be rapidly reversible in 1.5–2.2 h.^12^ Several studies have evaluated the efficacy of tirofiban in patients receiving EVT.^13^, ^14^ However, fewer studies have focused on the factors affecting the outcomes of the EVT followed by tirofiban in AIS patients. In this study, we performed exploratory analyses regarding factors influencing functional outcomes in AIS patients who received adding tirofiban to EVT.

## 2 METHODS

### 2.1 Study design and patient selection

In this retrospective study, consecutive AIS patients treated with EVT followed by tirofiban from January 2017 to October 2021 in Xuanwu Hospital, Capital Medical University, were enrolled. This study was approved by the Ethics Committees of Xuanwu Hospital (NO. 2017030) and was conducted according to the principles of the Declaration of Helsinki. All participants or their legal representatives signed informed consent forms for data collection. Additional written informed consent for tirofiban off‐label use was obtained before the use of tirofiban.

The inclusion criteria were as follows: age ≥18 years old; establishing a clinical diagnosis of AIS; received EVT according to the guidelines^15^, ^16^; no intracranial hemorrhage (ICH) indicated in brain CT immediately after EVT; treated with tirofiban following EVT. The exclusion criteria were as follows: missing National Institutes of Health Stroke Scale (NIHSS) scores and other critical information; received intravenous thrombolysis (IVT), including but not limited to rt‐PA, urokinase, and tenecteplase; received angiography only; contraindications of tirofiban at presentation; loss of follow‐up at 90 days.

### 2.2 Endovascular treatment procedures

Skilled interventionalists performed the EVT procedures. All procedures were performed under local or general anesthesia with/without sedative agents. All patients received percutaneous femoral artery puncture, and an 8‐F sheath was inserted. After anticoagulation with intra‐arterial (IA) heparin (80 U/kg), initial angiography was performed from the guiding catheter. Once the occlusion was confirmed, EVT was performed. The thrombectomy device and intervention strategies were at the discretion of the interventionists. To minimize the risk of endothelial damage, the procedure was attempted a maximum of three times. If EVT failed to fully recanalize the occluded artery, rescue treatment with emergency stenting or balloon angioplasty would be conducted.

### 2.3 Administration of Tirofiban

During the EVT procedure, whether a bolus injection of tirofiban (Tirofiban Hydrochloride and Sodium Chloride Injection, Grand Pharmaceutical, Co, Ltd, China) via intravenous or intra‐arterial pathway was at the interventionalists' discretion. The routine practice for intra‐arterial or intravenous injection of tirofiban was a bolus dose of 10 μg/kg. Once ICH was ruled out based on post‐endovascular head CT without contrast, an intravenous infusion of tirofiban was continuously administered at a rate of 0.1 μg/kg/min. The clinicians adjusted the dose and duration of tirofiban according to the patient's status (neurological function, bleeding, etc.). Four hours before the end of infusion, an oral antiplatelet agent (aspirin 100 mg and/or clopidogrel 75 mg) was given as bridging therapy.

### 2.4 Data collection

Patient demographic and clinical data were collected retrospectively. Demographic data included age, gender, body mass index (BMI), previous history of stroke, risk factors (such as hypertension, diabetes, hyperlipidemia, coronary heart disease, and smoking), and baseline medication.

Clinical data included tirofiban treatment information, EVT procedure information, and etiology of stroke based on the Trial of ORG 10172 in Acute Stroke Treatment (TOAST).^17^ Neurological deficit was measured by NIHSS (scores of 0–42),^18^ and functional outcome was measured with a modified Ranking Scale (mRS, 0 [no symptoms] to 6 [death]).^19^ The NIHSS was evaluated on admission, post‐EVT, 24‐ and 72‐h after onset, pre‐, and post‐tirofiban treatment, and at discharge. Early neurological recovery was defined as a reduction of at least 8 points on the NIHSS score or NIHSS 0–1 in 72 h.^20^ A reduction of at least four points of NIHSS after tirofiban treatment compared with that before tirofiban treatment was defined as an effective response to tirofiban.^21^ The mRS before the stroke and 90 days after the stroke were assessed. The mRS at 90 days after EVT was dichotomized into two variables: favorable outcome (mRS 0–2) and unfavorable/poor outcome (mRS 3–6).^22^ Furthermore, an excellent recovery was defined as an mRS 0–1.^20^ Laboratory data on admission, including complete blood count, serum lipid levels, serum glucose level, fibrinogen, and D‐Dimer were also recorded. Alberta Stroke Program Early Computed Tomography Score (ASPECTS) was assessed from admission non‐contrast‐enhanced CT images.^23^ The brain computed tomographic arteriography (CTA) on admission was used to evaluate the occlusion site. Non‐contrast enhanced CT was performed immediately and 24 h after EVT or at any time when symptoms got worsened. Transcranial color doppler (TCD) ultrasound and/or CTA were performed within 72 h to determine whether reocclusion of the recanalized arteries existed or not, which was defined as initially complete or partially recanalized arteries with occlusion recurring at the same site.^24^ Recanalization status was evaluated by the modified treatment in cerebral infarction (mTICI) score. Successful recanalization was defined as an mTICI 2b‐3 and unsuccessful recanalization was defined as an mTICI of 0‐2a^25^

### 2.5 Outcome evaluation

Efficacy outcomes: mRS at 90 days and early neurological recovery at 72 h.

Safety outcomes: Symptomatic ICH (sICH) was defined according to the definition of the ECASS‐III study^26^: any extravascular blood in the brain or within the cranium that led to an increase of four points or more on NIHSS or death, which was identified as the predominant cause of the neurological deterioration.

### 2.6 Statistical analysis

Statistical analyses were performed with SPSS version 26.0 (IBM Corp). Data were presented as the mean (standard) deviation for continuous variables and as counts and percentages (%) of subjects for categorical variables. The Kolmogorov–Smirnov test for normality and the equal variance test was performed for continuous variables. Data that do not exhibit a normal distribution were analyzed via a nonparametric equivalent. Univariate comparison of two groups was performed with a two‐sided Student's *t*‐test or the Mann–Whitney rank sum test for continuous variables and with Pearson's χ^2^ or Fisher test for categorical variables. Univariate analysis was performed to find the significant factors (*p* < 0.1). The risk factors from the univariate analysis and potential confounders that might affect the outcomes were added to multivariate logistic regression models. ROC curve and area under the curve (AUC) were used to evaluate the specificity and sensitivity of factors to predict the outcome and the optimal cut‐off value (Youden index). All the results with *p* < 0.05 were considered to have a significant statistical difference.

## 3 RESULTS

### 3.1 Characteristics of the patients

**FIGURE 1:**
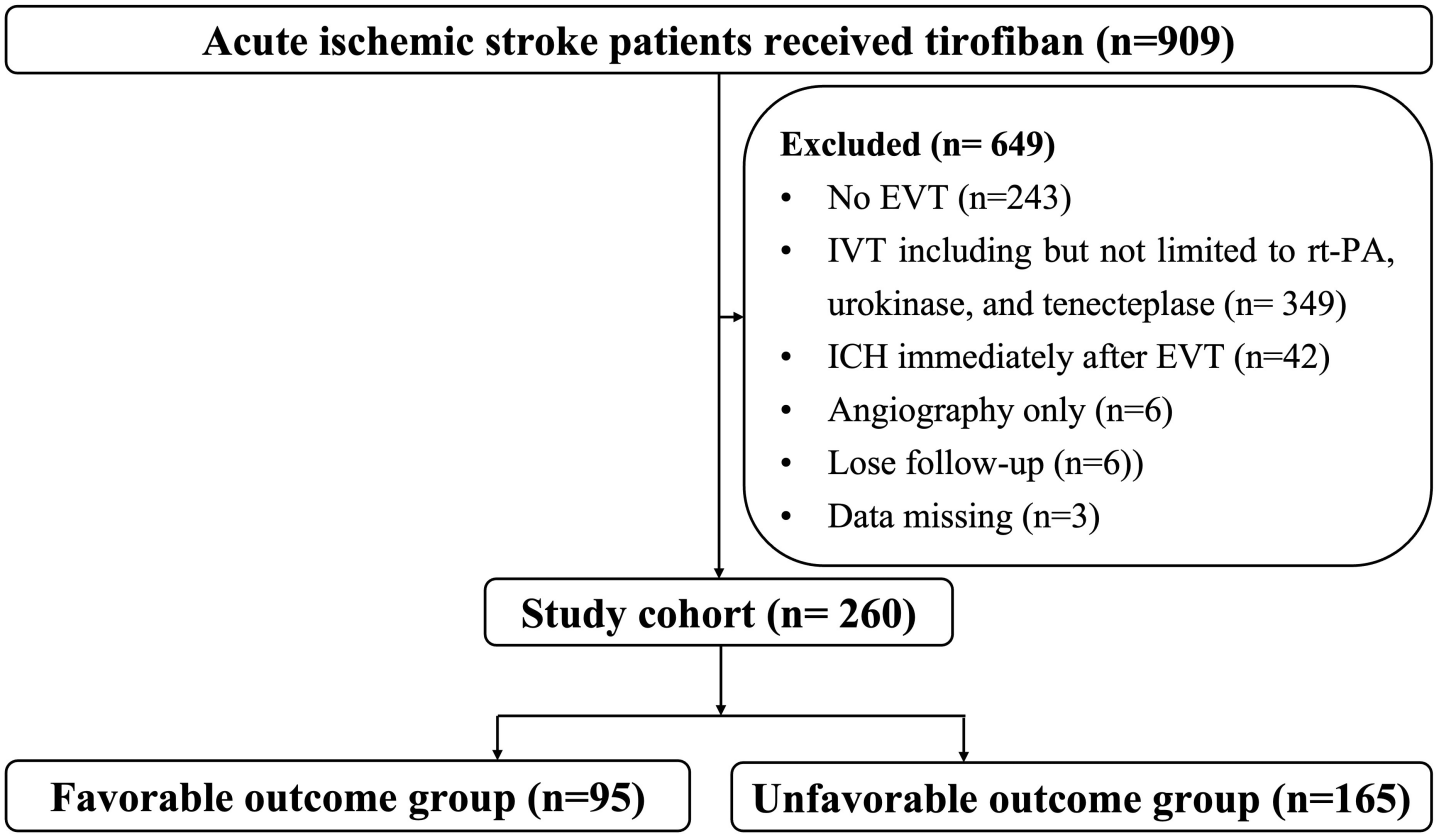
Flow diagram. This figure shows the enrollment information of patients, including the reasons for exclusion and the grouping situation

From January 2017 to October 2021, a total of 260 consecutive AIS patients who received EVT followed by tirofiban were enrolled (Figure 1). Among the 260 patients, the average age was 63.0 ± 11.7 years old, and 73.8% of them (192 out of 260) were male. The median ASPECTS was 9 (IQR 8–10). A total of 138 (53.1%) patients had occlusion in the anterior circulation and 109 (41.9%) patients suffered from posterior circulation occlusion. According to the TOAST classification, a total of 211 (81.5%) patients were diagnosed with large‐artery atherosclerosis (LAA) and 42 (16.2%) patients were diagnosed with cardio‐embolism (CE). The median onset to puncture time (OPT) was 444 (IQR 317, 667) min, and the median onset to reperfusion (ORT) time was 526 (IQR 400, 735) min. The average number of passes (NOP) was 0.93. The median time between symptom onset and the start of tirofiban treatment was 568 (IQR 428, 801) min. The median of NIHSS on admission was 17 (IQR 12, 25), decreasing to 15 (IQR 6, 22.75) post‐tirofiban treatment, and finally to 11 (IQR 4, 19) at discharge. The medical therapies, laboratory examinations, pre‐stroke status, and surgical details are summarized in Table 1.

**TABLE 1:** Characteristics of patients

	All patients (*n* = 260)	Favorable outcome (*n* = 95)	Unfavorable outcome (*n* = 165)	*p* value
Age, median (SD), years	63 (11.70)	61 (10.84)	64 (12.07)	0.054
Male, *n* (%)	192 (73.8)	77 (81.1)	115 (69.7)	**0.045**
BMI, median (SD), kg/m^2^	25.7 (3.9)	26.0 (4.2)	25.5 (3.6)	0.346
SBP	151 (22.6)	148 (20.0)	153 (23.9)	0.092
DBP	86 (14.2)	85 (13.9)	87 (14.3)	0.553
NIHSS on admission, median (P25, P75)	17 (12, 25)	12 (9, 19)	20 (14.25, 30)	**<0.001**
ASPECT, median (P25, P75)	9 (8, 10)	9 (8, 10)	9 (8, 10)	0.418
Medical history, *n* (%)				
Hypertension	192 (73.8)	63 (66.3)	129 (78.2)	**0.036**
Diabetes mellitus	79 (30.4)	29 (30.5)	50 (30.3)	0.970
Coronary heart disease	50 (19.2)	14 (14.7)	36 (21.8)	0.163
Atrial fibrillation	36 (13.8)	11 (11.6)	25 (15.2)	0.422
Smoking (recent or current)	112 (43.1)	51 (53.7)	61 (37.0)	**0.009**
Ischemic stroke	71 (27.3)	21 (22.1)	50 (30.3)	0.153
Hemorrhagic stroke	7 (2.7)	1 (1.1)	6 (3.6)	0.400
Lower extremity DVT	1 (0.4)	0 (0)	1 (0.6)	1.000
Antiplatelet drugs	64 (24.6)	18 (18.9)	46 (27.9)	0.107
Anticoagulant	13 (5)	5 (5.3)	8 (4.8)	1.000
Lipid‐lowering drugs	43 (16.5)	16 (16.8)	27 (16.4)	0.920
Clinical data				
Leukocyte, mean (SD), 10^12^/L	10.0 (3.5)	9.2 (2.9)	10.4 (3.7)	**0.005**
NEUT, mean (SD), %	77.8 (15.8)	76.1 (11.5)	78.8 (17.8)	0.171
PLT, mean (SD), 10^9^/L	223 (58.0)	224 (55.2)	222 (59.7)	0.778
Erythrocyte, mean (SD), 10^12^/L	4.6 (0.7)	4.6 (0.6)	4.5 (0.7)	0.294
Hemoglobin, mean (SD), g/L	139 (21.1)	143 (19.4)	137 (21.8)	0.060
Glucose, mean (SD), mmol/L	8.6 (3.6)	7.2 (2.7)	9.3 (3.9)	**<0.001**
TG, mean (SD), mmol/L	1.4 (0.9)	1.4 (0.9)	1.4 (1.0)	0.747
T‐Chol, mean (SD), mmol/L	4.5 (1.5)	4.5 (1.1)	4.5 (1.6)	0.814
LDL‐c, mean (SD), mmol/L	2.8 (0.9)	2.8 (0.9)	2.7 (1.0)	0.427
Fib, mean (SD), mmol/L	3.5 (11)	3.28 (0.9)	3.7 (1.1)	**0.004**
D‐dimer, mean (SD), mmol/L	1.7 (3.1)	0.9 (2.2)	2.1 (3.5)	**<0.0011**
TOAST, *n* (%)				
LAA	211 (81.5)	77 (81.1)	134 (81.2)	0.811
CE	42 (16.2)	16 (16.8)	26 (15.8)	0.819
ODC	3 (1.2)	2 (2.1)	1 (0.6)	0.556
UND	4 (1.5)	0 (0)	4 (2.4)	0.300
Infarction area, *n* (%)				
AC	138 (53.1)	60 (63.2)	78 (47.3)	**0.013**
PC	109 (41.9)	29 (30.5)	80 (48.5)	**0.005**
AC & PC	12 (4.6)	5 (5.3)	7 (4.2)	0.706
EVT				
OPT, median (P25, P75), min	444 (317, 667)	433 (271, 605.5)	452 (346.3691.5)	0.223
ORT, median (P25, P75), min	526 (400, 735)	505 (364, 682)	539 (421, 766.8)	0.124
General anesthesia, *n* (%)	89 (34.2)	15 (16.0)	74 (44.8)	**<0.001**
Stent retriever, *n* (%)	165 (55.4)	55 (57.9)	110 (66.7)	0.157
Aspiration, *n* (%)	186 (71.5)	72 (75.8)	114 (69.1)	0.249
Stent implantation, *n* (%)	103 (39.6)	39 (41.1)	64 (38.8)	0.719
Balloon dilatation, *n* (%)	119 (45.8)	37 (38.9)	82 (49.7)	0.094
Artery thrombolysis, *n* (%)	14 (5.4)	4 (4.2)	10 (6.1)	0.517
NOP, mean (SD), times	0.93 (0.93)	0.78 (0.81)	1.02 (0.98)	**0.039**
mTICI ≥2b, *n* (%)	243 (93.5)	94 (98.9)	149 (90.3)	**0.007**
Residual stenosis after recanalization, *n* (%)	78 (30)	28 (29.5)	50 (30.3)	0.888
Treatment of tirofiban				
EVT to Tirofiban, median (P25, P75), min	0 (0, 49.5)	0 (0, 42)	0 (0, 49.5)	0.561
Onset to Tirofiban, median (P25, P75), min	568 (428, 801)	564 (455, 780)	571.5 (420, 837.75)	0.674
Arterial injection, *n* (%)	60 (23.1)	22 (23.2)	38 (23.0)	0.981
Duration of Tirofiban >24 h, *n* (%)	79 (30.4)	57 (60)	22 (13.3)	**<0.001**
Effective response to tirofiban, *n* (%)	103 (39.6)	71 (74.7)	32 (19.4)	**<0.001**
NIHSS at a different time points, mean (SD), points				
After EVT	16 (9, 23.75)	7 (4, 12)	20 (15, 30)	**<0.001**
Before the treatment of tirofiban	17.5 (12, 25)	12 (8, 18)	20 (15, 31)	**<0.001**
At the end of tirofiban treatment	15 (6, 22.75)	5 (2, 9)	20 (14.25, 30)	**<0.001**
NIHSS at 72 h	14 (5.25, 23)	4 (2, 8)	20 (14, 30)	**<0.001**
NIHSS at discharge	11 (4, 19)	3 (1, 5)	16 (11, 28)	**<0.001**
Outcomes, *n* (%)				
Early neurological recovery at 72 h	76 (29.2)	56 (58.9)	20 (12.1)	**<0.001**
Reocclusion within 72 h	25 (9.6)	2 (2.1)	23 (13.9)	**0.002**
sICH	16 (6.2)	1 (1.1)	15 (9.1)	**0.003**

Abbreviations: AC, anterior circulation; ASPECT, Alberta Stroke Program Early CT Score; BMI, body mass index; CE, cardio‐embolism; DBP, diastolic blood pressure; EVT, endovascular treatment; Fib, fibrinogen; HCY, homocysteine; LAA, large artery atherosclerosis; LDL‐C, low‐density lipoprotein cholesterol; mTICI, modified Thrombolysis in Cerebral Infarction Score; NEUT, neutrophil; NIHSS, National Institute of Health stroke scale; NOP, number of passes; ODC, other determined cause; OPT, onset to puncture time; ORT, onset to reperfusion time; PC, posterior circulation; PLT, platelet; SBP, systolic blood pressure; sICH, symptomatic cerebral hemorrhage; T‐Chol, total‐cholesterol; TG, triglyceride; TOAST, the trial of Org 10172 in acute stroke treatment; UND, undetermined cause. Significance of bold values: *p* < 0.05.

### 3.2 Outcomes of patients

**FIGURE 2:**
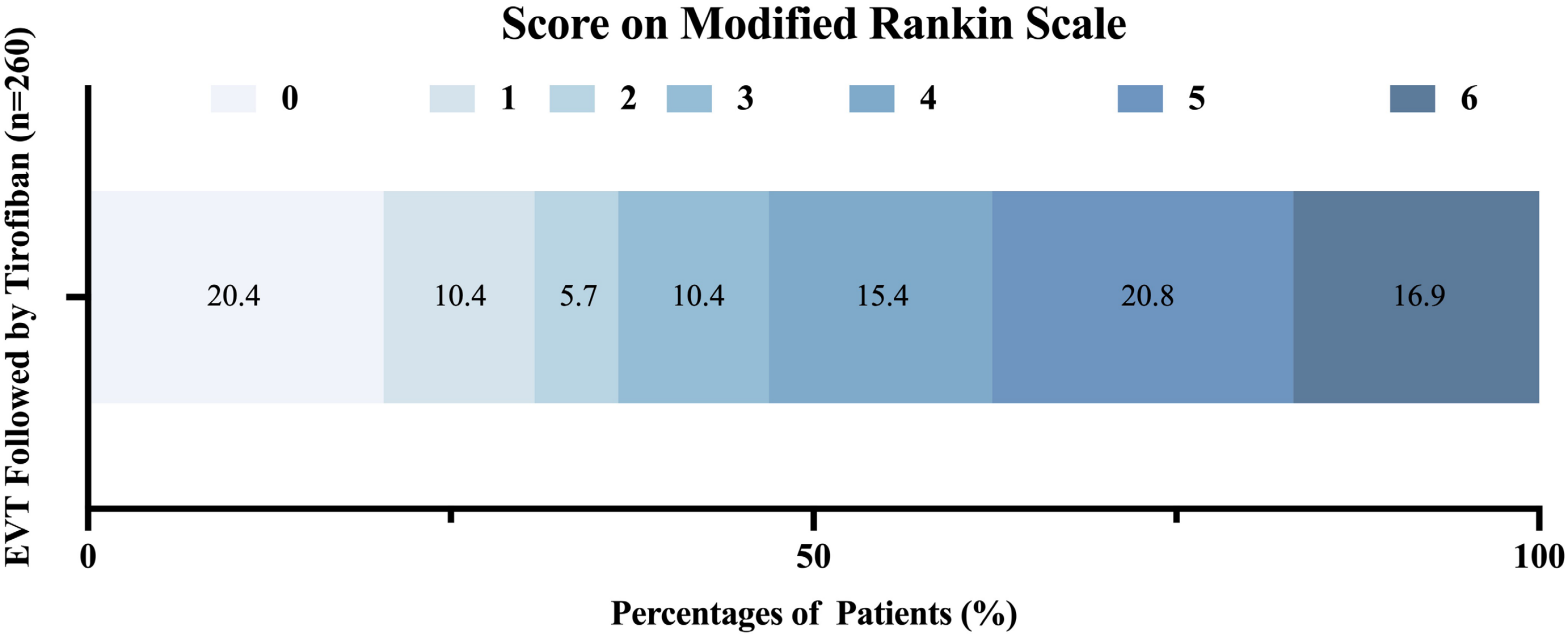
Proportion distribution of different scores on mRS in AIS patients who received EVT followed by tirofiban treatment

A total of 95 patients (36.5%) achieved a favorable outcome (mRS 0–2) at 90 days (Figure 2), in comparison to the 32.6–71.4% demonstrated in the five large randomized trials (RCTs, the MR CLEAN, ESCAPE, EXTEND‐IA, SWIFT‐PRIME, and REVASCAT studies), and 30.8% of patients achieved the excellent recovery (mRS 0–1) (Table 2). Early neurological improvement at 72 h after treatment occurred in 79 (29.2%) patients. Two hundred and forty‐three (93.5%) patients achieved successful recanalization (mTICI 2b‐3), which was superior to the mean rate (71%) reported in the five RCTs. Twenty‐five patients (9.6%) were found with vessel reocclusion within 72 h after EVT. The event of sICH occurred in 16 patients (6.2%), and 44 patients (16.9%) died 90 days after stroke onset, similar to the range in the five large, randomized trials (Table 2).^27^

**TABLE 2:** Clinical outcomes of all patients versus five randomized controlled trials

	All patients (*n* = 260)	Five RCTs	Meta‐analysis of five RCTs (*n* = 634)
Efficacy outcomes			
90‐day mRS ≤2	36.5%	32.6–71.4%	46%
90‐day mRS ≤1	30.8%	–	26.9%
mTICI ≥2b	93.5%	58.7–86%	71%
Reocclusion at 72 h	9.6%	–	–
Safety outcomes			
sICH	6.2%	0–7.7%	4.4%
Mortality	16.9%	9–21%	15.3%

Abbreviations: mRS, modified Ranking Scale; mTICI, modified Thrombolysis in Cerebral Infarction; NIHSS, National Institute of Health stroke scale; RCT, randomized control trial; sICH, symptomatic intracranial hemorrhage.

### 3.3 Influencing factors

According to the mRS score, patients were divided into two groups: patients with favorable outcomes and patients with unfavorable outcomes (Table 1). There was no significant difference between the two groups regarding age and BMI. A higher ratio of males (81.1% vs. 69.7%, *p* = 0.045) and a larger proportion of patients with recent or current smoking history (53.7% vs. 37%, *p* = 0.009) were found in the favorable outcome group. Compared to the unfavorable outcome group, patients in the favorable outcome group had a lower frequency of hypertension (66.3% vs. 78.2%, *p* = 0.036). In terms of laboratory examination, the level of leukocyte, glucose, fibrinogen, and D‐dimer in the favorable outcome group were lower than those in the unfavorable outcome group.

The favorable outcome group showed lower NIHSS on admission, post‐EVT, pre‐and posttreatment of tirofiban, at 72 h, and discharge. Patients in the favorable outcome group were more likely to suffer from occlusion in the anterior circulation than in the unfavorable outcome group (63.2% vs. 47.2%, *p* = 0.013). More patients in the unfavorable outcome group suffered general anesthesia (16.0% vs. 44.8%, *p* < 0.001). The percentage of successful recanalization was 98.9% in the patients with a favorable outcome, compared to 90.3% in those with an unfavorable outcome (*p* = 0.007). In the patients who experienced reocclusion, there were two (2.1%) patients in the favorable outcome group and 23 (13.9%) in the unfavorable outcome group (*p* = 0.002).

An effective response to tirofiban therapy was achieved by 74.4% of patients in the favorable outcome group and 19.4% of patients in the unfavorable outcome group (*p* < 0.001). Early neurological improvement at 72 h after stroke onset occurred in 58.9% of the favorable outcome group and 12.1% of the unfavorable outcome group. Fewer patients in the favorable outcome group had sICH compared to those in the unfavorable outcome group (1.1% vs. 9.1%, *p* = 0.003).

**FIGURE 3:**
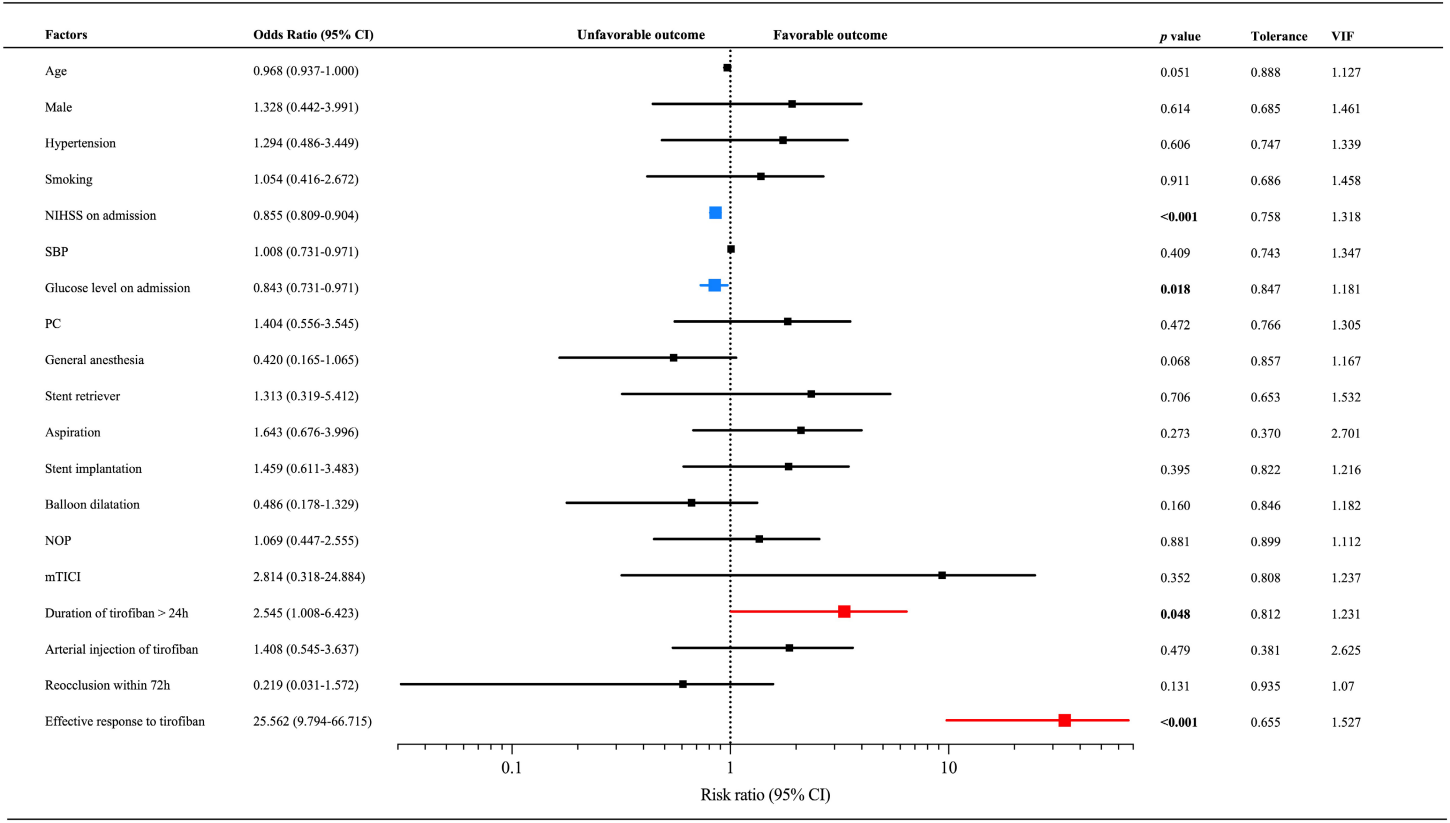
Binary logistic regression analysis of factors associated with favorable outcomes

Multivariate logistic regression was performed by enrolling the significant factors from the univariate regression analysis and potential confounders that might affect the outcomes to determine the influencing factors of the outcomes after EVT followed by tirofiban treatment (Figure 3). As shown in the forest plot, the duration of tirofiban >24 h (adjusted OR: 2.545; 95% CI: 1.008–6.423; *p* = 0.048) and effective response to tirofiban (adjusted OR: 25.562; 95% CI: 9.794–66.715; *p* < 0.001) were related to the favorable outcome. Higher NIHSS (adjusted OR: 0.855; 95% CI: 0.809–0.904; *p* < 0.001) and glucose level on admission (adjusted OR: 0.843; 95% CI: 0.731–0.971; *p* = 0.018) were associated with unfavorable outcomes. Considering the difference in NIHSS between the two groups in baseline, multicollinearity tests were performed among covariables, which demonstrated that the effect of multicollinearity among independent variables of each factor is not significant (Tolerance >0.1 and VIF <5) (Figure 3).

In addition, we analyzed the factors that influence short‐term effective responses to tirofiban. A total of 103 patients had an effective response to tirofiban. Compared to those without an effective response to tirofiban, patients with an effective response to tirofiban suffered from a lower frequency of hypertension and hemorrhagic stroke, and also had lower systolic blood pressure and diastolic blood pressure at admission. Regarding laboratory examination, patients with an effective response to tirofiban had a lower level of leukocyte, glucose, and fibrinogen. Furthermore, patients with an effective response to tirofiban showed a lower ratio of general anesthesia, a higher ratio of successful recanalization, and a shorter reperfusion time. Patients with an effective response to tirofiban experienced fewer reocclusion within 72 h and sICH (Table S1). Further multivariate logistic regression showed that higher glucose level on admission was related to the noneffective response to tirofiban (Figure S1).

**FIGURE 4:**
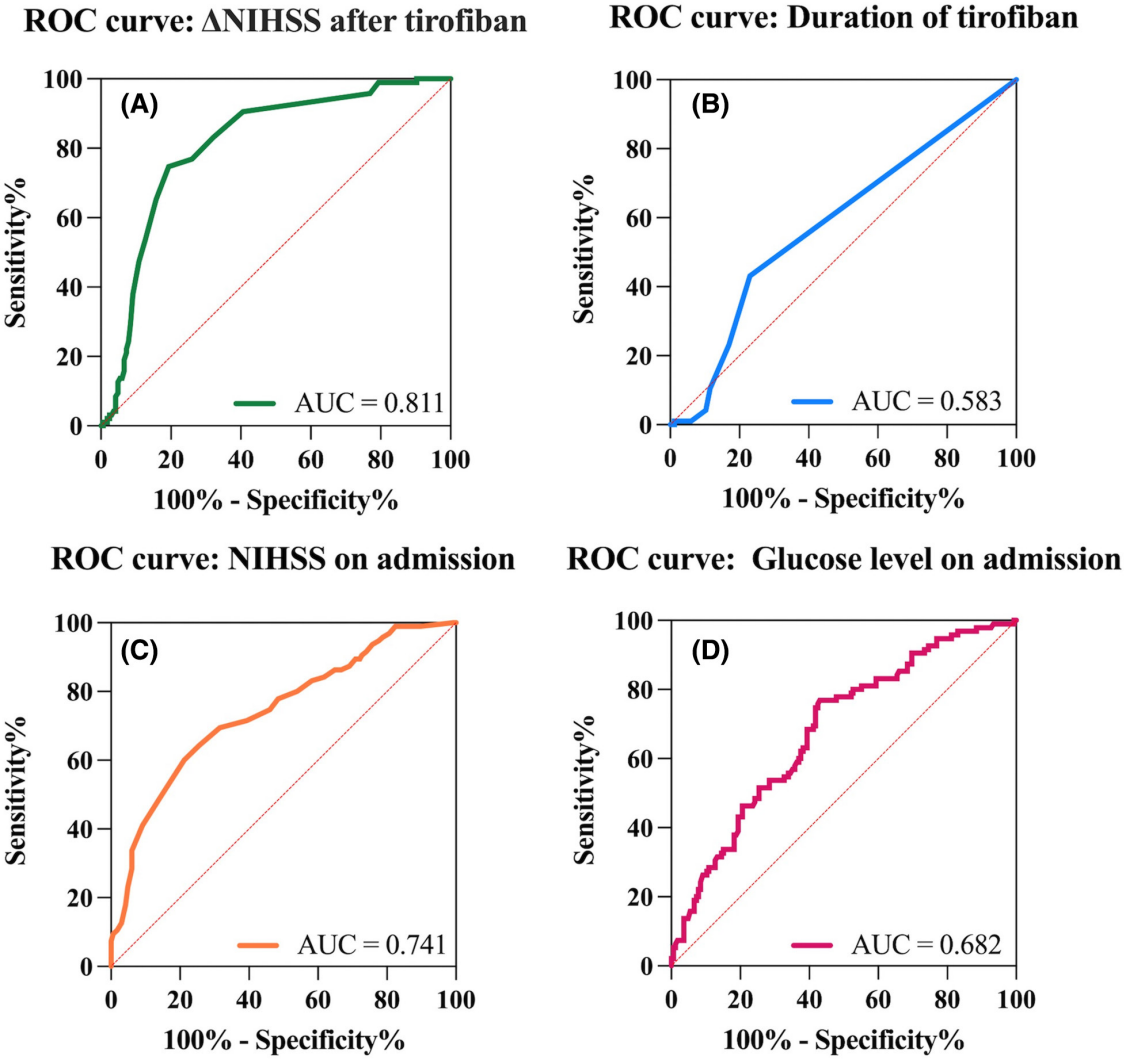
ROC curves for independent factors that influence the favorable outcome. (A) ROC curve for ΔNIHSS after tirofiban; (B) ROC curve for the duration of tirofiban; (C) ROC curve for NIHSS on admission; and (D) ROC curve for glucose level on admission

The AUC to predict a 90‐day favorable outcome was 0.811 (95% CI: 0.756–0.865, *p* < 0.001) for change of NIHSS pre‐ and post‐tirofiban treatment, 0.583 (95% CI: 0.510–0.655, *p* = 0.026) for the duration of tirofiban, 0.741(95% CI: 0.678–0.804, *p* < 0.001) for NIHSS on admission, and 0.682 (95% CI: 0.616–0.748, *p* < 0.001) for glucose level on admission (Figure 4). The cut‐off value of reduction of NIHSS after tirofiban treatment compared with that before tirofiban treatment, duration of tirofiban, NIHSS on admission, and glucose level on admission to predict the 90‐day favorable outcome was 3.5 points (74.7% sensitivity, 80.6% specificity), 36 h (43.2% sensitivity and 77.0% specificity), 13.5 points (60% sensitivity and 78.8% specificity), and 7.91 mmol/L (76.8% sensitivity and 57.0% specificity).

## 4 DISCUSSION

The five RCTs have demonstrated the utility and benefit of EVT for AIS patients due to LVO.^28^, ^29^, ^30^, ^31^, ^32^ However, thrombi retrieved from patients with acute artery occlusion were highly heterogeneous. The side effect of EVT was endothelial injuries, and following platelet aggregation, which may cause thromboembolic complications and early reocclusion. Tirofiban reversibly and selectively competes against GPIIb/IIIa receptors on platelets and could inhibit platelet aggregation within 5 min after intravenous injection,^33^ of which the peak time was less than 30 min and the stable plasma concentration could be achieved within 1 h.^34^ Recent studies have indicated that tirofiban combined with EVT appeared to be safe and potentially effective in treating AIS.^35^, ^36^, ^37^ However, data regarding influencing factors for the prognosis of AIS patients treated with EVT followed by tirofiban were still scarce.

In a retrospective cohort study using data prospectively collected from the US nationwide Get With The Guidelines‐Stroke (GWTG‐Stroke) registry, Jahan et al^38^ found that in routine clinical practice, the median pretreatment NIHSS score among AIS patients who underwent endovascular reperfusion therapy was 17 (IQR 12–22), of which 85.9% achieved substantial reperfusion (mTICI 2b‐3), 39.8% had functional independence (mRS 0–2), and 28.7% achieved disability‐free (mRS 0–1) at 3‐month follow‐up. Adverse events were sICH in 6.7% of patients and in‐hospital mortality/hospice discharge in 19.6% of patients. Tirofiban was found to be important in sustaining the thrombolytic process and preventing reocclusion after EVT, especially inhibiting the “no‐reflow phenomenon” in microcirculation.^39^ Compared with previous studies,^35^, ^36^ EVT combined with tirofiban in this study did not increase the risk of sICH (6.2%) and mortality (16.9%) of AIS patients. The proportion of successful recanalization (mTICI 2b‐3) in patients in this study has been up to a higher ratio of 93.5%, which was similar to Cheng's study (92.6%),^40^ and the ratio of reocclusion within 72 h after EVT was 9.2%, which was similar to Yan's study (10.4%).^41^ Thus, tirofiban seemed to be effective and safe to inhibit fresh thrombosis and maintain vascular recanalization after EVT. Furthermore, we found that patients with a favorable outcome tended to have a higher rate of successful recanalization and a lower reocclusion rate within 72 h after EVT, indicating that improving and maintaining recanalization might be a pivotal point influenced by tirofiban, which is related to short‐term and long‐term neurological recovery.

Additionally, the potential of tirofiban to ameliorate cerebral blood flow (CBF) should be considered, which was expected to be helpful to elucidate the mechanisms for the effective response to tirofiban. A significant number of AIS patients who underwent EVT with large vessel recanalization also had poor functional outcomes, of which impaired microvascular reperfusion played a major role.^42^ Recent studies have shown that favorable collateral status predicted less cerebral edema, whereas the embarrassed microcirculation and collateral compensatory capacity were related to larger cerebral infarction,^43^, ^44^ which could affect the functional outcomes both in patients with small subcortical infarct or LVO.^45^, ^46^ Yang et al found that as an adjunct to EVT, IV tirofiban increased recanalization from 64.1% to 96.7% as compared with the control group, with an increased rate of 3‐month good outcome (41.4% vs. 12.2%).^47^ Thus, tirofiban which can facilitate the recanalization rate and reperfusion process is suitable for patients with incomplete recanalization and non‐recanalization after EVT.^48^, ^49^ In this study, we found that tirofiban reduced the incidence of arterial reocclusion in 72 h after EVT, which is related to the maintenance of cerebral perfusion. Improvement of CBF by tirofiban would contribute to a favorable outcome.

As gradually expanding studies investigate the safety and efficacy in patients who underwent EVT followed by tirofiban, there is an increasing need to analyze which subgroups of patients are most likely to benefit from EVT combined with tirofiban. The female ratio, baseline NHISS score, the level of leukocyte, glucose, fibrinogen, and D‐dimer at admission, the rate of occlusion in the posterior circulation, number of passes during the EVT, and general anesthesia ratio of patients in the favorable outcome group were lower than those in the unfavorable group. NIHSS and glucose level on admission were independent predictors for an unfavorable outcome.

Several studies have reported gender differences in the field of ischemic stroke, and women seem more likely to have atypical symptoms and worse functional outcomes.^50^ In regards to the baseline NIHSS, similar results were reported by Cheng et al., where a low‐onset NIHSS score was a good predictor of good outcomes after endovascular recanalization.^51^ Rinkel et al^52^ found that hyperglycemia on admission (≥7.8 mmol/L) is associated with worse functional outcomes and an increased risk of sICH after EVT, which was consistent with our study. An interesting phenomenon was observed in this study: patients in the favorable outcome group had a higher rate of smoking than that in the unfavorable group. This paradox also appeared in studies evaluating the clinical outcomes of clopidogrel in AIS patients,^53^ which related to the altered pharmacokinetics of clopidogrel. Current or recent smoking can induce the activity of cytochrome P450 in liver cells, and thus, promote the conversion of clopidogrel into its active metabolite leading to more potent efficacy.^53^ Differing from clopidogrel, tirofiban binds primarily to plasma proteins and is metabolized by the kidney, hence, there is a prolonged half‐life of tirofiban in patients with renal insufficiency. Whether smoking affected tirofiban pharmacokinetics is unclear and it might require further evidence.

The proportion of effective response to tirofiban and duration of tirofiban exceeding 24 h were higher in the favorable outcome group than those in the unfavorable group. Duration of tirofiban >24 h and effective response to tirofiban were related to a favorable outcome. Previous studies have reported that reocclusion of recanalized vessels continues to occur up to 24 h after endovascular therapy, and continuous intravenous tirofiban could decrease reocclusion at 24 h after IVT. Thus, continuous intravenous tirofiban for a full 24 h may be more effective to prevent early reocclusion of the recanalized vessel after EVT.^40^ Thrombectomy combined with tirofiban was safe for arterial reocclusion prevention, but care should be taken regarding drug doses and duration. Zhu et al^54^ indicated that age >70 years, NIHSS >15, and overall dose >10 mg are risk factors for ICH events with tirofiban usage. It is recommended that the dose for intravenous pumping of tirofiban is 0.1–0.2 μg/(kg/min), which should be adjusted according to the changes in the patient's renal function and neurological function. The duration of tirofiban is advised as 12–24 h after EVT therapy, which can be extended to 24–48 h post‐EVT for patients with repeated occlusion due to endothelial injury.^55^ Our study indicated that the optimal cut‐off value to predict neurological function independence (favorable outcome) was 36 h, which indicated that appropriately extending the duration of tirofiban on the base of recommendation would be associated with a better functional outcome. However, it still needed further supported evidence. We also found that the efficacy of tirofiban in the acute phase affected long‐term prognosis, and patients with short‐term effective treatment response to tirofiban tend to have a better long‐term favorable outcome. Chalos et al.^56^ reported that the NIHSS within 1 week satisfied the requirements for a surrogate endpoint and may be used as an alternative primary outcome measure for ischemic stroke. A reduction of NIHSS equal to 3.5 after tirofiban treatment would be the optimal diagnostic power to predict a favorable outcome. And the higher glucose level on admission might be a predictor for a noneffective response to tirofiban. The relationship and underlying mechanism between glucose level and the efficacy of tirofiban still need further verification.

However, there were some limitations in this study. Firstly, the number of patients analyzed in this study relatively small, and the optimal strategy for tirofiban administration after EVT still needs further large‐sample and multicenter studies. Secondly, this was a retrospective cohort study. Although regression analysis was used to correct the factors that might have a mismatch, there were still some confounding biases, such as information bias brought by follow‐up. Thirdly, patients who received IVT in this study were excluded. Although the current studies suggested that there was no significant difference in the effect of bridging therapy and direct thrombectomy on functional outcomes,^57^ whether the combination of thrombolytic drugs and tirofiban is effective and safe in patients with EVT remains to be validated. Finally, the postoperative CBF and collateral status, which predict the prognosis of patients, were not routinely evaluated in this study. Advanced imaging techniques, such as perfusion CT, and arterial spin labeling MRI, should be warranted to evaluate cerebral perfusion to further explore the mechanism of tirofiban.

## 5 CONCLUSIONS AND FUTURE PERSPECTIVES

The results of this study suggested that tirofiban following EVT can effectively increase the rate of recanalization. An effective response to tirofiban was likely to be an independent predictor for neurological function independence at 90 days. Increased admission NIHSS and glucose level may adversely affect the outcome of EVT combined with tirofiban. The optimal duration of tirofiban treatment to achieve a good neurological outcome was 36 h, which indicated that a large‐sample randomized controlled trial was warranted to determine the optimal administration of tirofiban

## AUTHOR CONTRIBUTIONS

YW and HM contributed to the work equally and should be regarded as the first authors. YW, QM, and XJ designed the study. YW and HM drafted the manuscript. HM conducted the data analysis. QZ, FJ, and YX collected the data. All authors read and approved the final version of the manuscript. QM and XJ were the guarantor of the study and had full access to the data.

## CONFLICT OF INTEREST

The authors declare no conflict of interest.

## Supporting information


Figure S1.

Table S1.

## Data Availability

Detailed data are available from the corresponding author upon reasonable request.
