# Supplementary figures and images for: Factors affecting the outcomes of tirofiban after endovascular treatment in acute ischemic stroke: Experience from a single center
Source: CNS Neurosci Ther. 2023 Jan 4;29(3):957–67. doi: 10.1111/cns.14058 (PMC9928549; doi:10.1111/cns.14058)

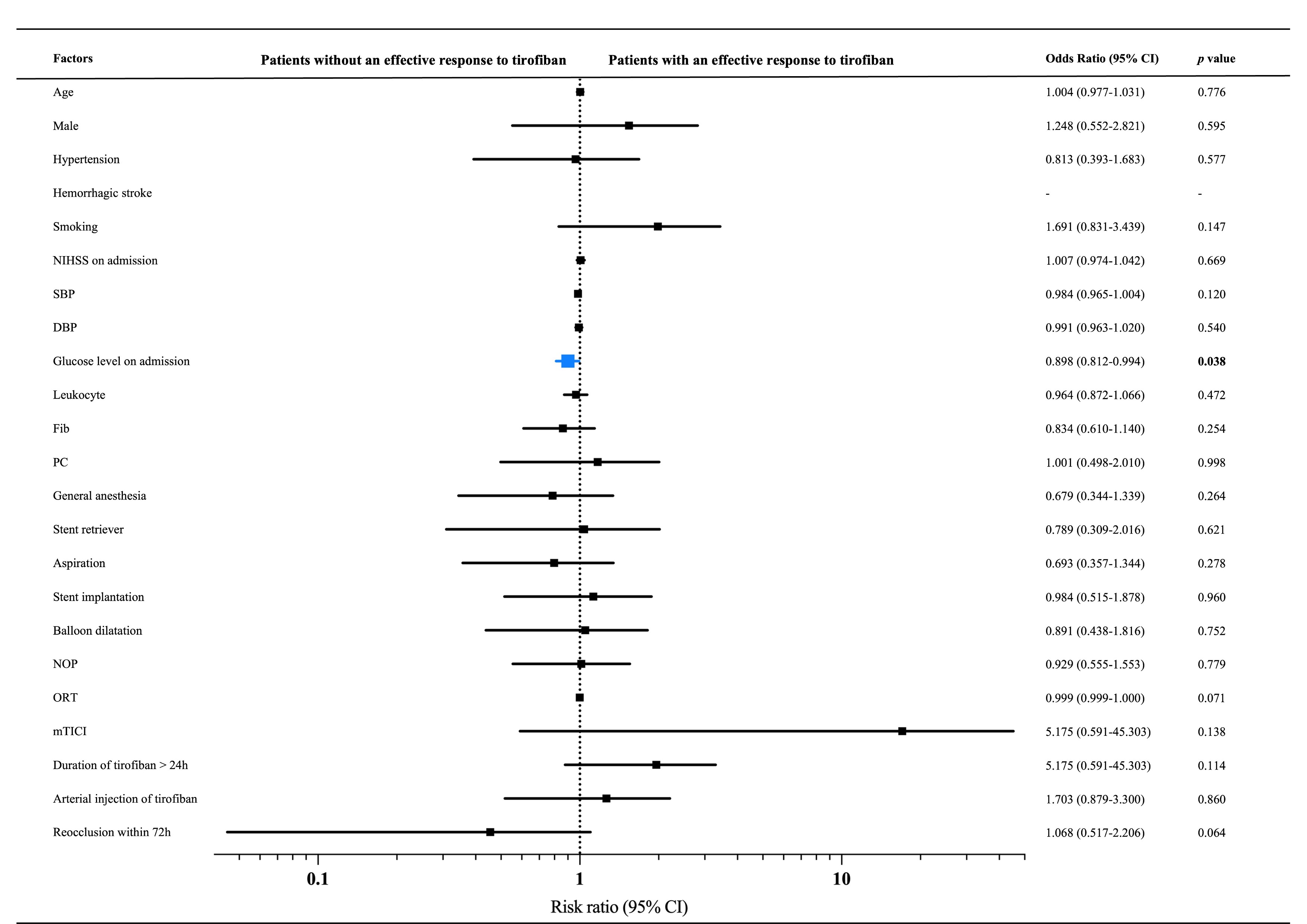

Supplement: Supplementary file 1 — Figure S1. [file CNS-29-957-s002.tiff]
