# Supplementary material for: Factors affecting the outcomes of tirofiban after endovascular treatment in acute ischemic stroke: Experience from a single center
Source: CNS Neurosci Ther. 2023 Jan 4;29(3):957–67. doi: 10.1111/cns.14058 (PMC9928549; doi:10.1111/cns.14058)
Supplement: Supplementary file 2 — Table S1. [file CNS-29-957-s001.docx]

## Supplementary Table 1. Characteristics of patients with or without effective response to tirofiban

|  | **All patients**  **(n=260)** | **Patients with an effective response to tirofiban (n=103)** | **Patients without an effective response to tirofiban (n=157)** | ***p* value** |
| --- | --- | --- | --- | --- |
| Age, median (SD), y | 63 (11.70) | 62 (11.5) | 63 (11.8) | 0.772 |
| Male, n (%) | 192 (73.8) | 80 (77.7) | 112 (71.3) | 0.256 |
| BMI, median (SD), kg/m^2^ | 25.7 (3.9) | 25.6 (4.1) | 25.8 (3.7) | 0.735 |
| SBP | 151 (22.6) | 144 (20.9) | 156 (22.6) | **< 0.001** |
| DBP | 86 (14.2) | 84 (14.5) | 88 (13.8) | **0.020** |
| NIHSS on admission, median (P25, P75) | 17 (12, 25) | 17 (12, 26) | 17 (12, 25) | 0.672 |
| ASPECT, median (P25, P75) | 9 (8, 10) | 9 (8, 10) | 9 (8, 10) | 0.527 |
| **Medical history, n (%)** | | | | |
| Hypertension | 192 (73.8) | 66 (64.1) | 126 (80.3) | **0.004** |
| Diabetes mellitus | 79 (30.4) | 26 (25.2) | 53 (33.8) | 0.144 |
| Coronary heart disease | 50 (19.2) | 16 (15.5) | 34 (21.7) | 0.221 |
| Atrial fibrillation | 36 (13.8) | 11 (10.7) | 25 (15.9) | 0.231 |
| Smoking (recent or current) | 112 (43.1) | 52 (50.5) | 60 (38.2) | **0.051** |
| Ischemic stroke | 71 (27.3) | 27 (26.2) | 44 (28) | 0.748 |
| Hemorrhagic stroke | 7 (2.7) | 0 (0) | 7 (4.5) | **0.044** |
| Lower extremity DVT | 1 (0.4) | 0 (0) | 1 (0.6) | 1.000 |
| Antiplatelet drugs | 64 (24.6) | 24 (23.3) | 40 (25.5) | 0.690 |
| Anticoagulant | 13 (5) | 3 (2.9) | 10 (6.4) | 0.211 |
| Lipid-lowering drugs | 43 (16.5) | 16 (15.5) | 27 (17.2) | 0.724 |
| **Clinical data** | | | | |
| Leukocyte, mean (SD), *10^12^/L | 10.0 (3.5) | 9.3 (2.9) | 10.4 (3.7) | **0.010** |
| NEUT, mean (SD), % | 77.8 (15.8) | 76.7 (14.3) | 78.6 (16.6) | 0.357 |
| PLT, mean (SD), *10^9^/L | 223 (58.0) | 219 (51.6) | 225 (61.7) | 0.439 |
| Erythrocyte, mean (SD), *10^12^/L | 4.6 (0.7) | 4.6 (0.6) | 4.5 (0.7) | 0.668 |
| Hemoglobin, mean (SD), g/L | 139 (21.1) | 141 (20.4) | 138 (21.4) | 0.200 |
| Glucose, mean (SD), mmol/L | 8.6 (3.6) | 7.5 (3.1) | 9.2 (3.8) | **< 0.001** |
| TG, mean (SD), mmol/L | 1.4 (0.9) | 1.4 (0.9) | 1.4 (1.0) | 0.790 |
| T-Chol, mean (SD), mmol/L | 4.5 (1.5) | 4.6 (1.9) | 4.5 (1.0) | 0.542 |
| LDL-c, mean (SD), mmol/L | 2.8 (0.9) | 2.7 (1.0) | 2.8 (0.9) | 0.421 |
| Fib, mean (SD), mmol/L | 3.5 (11) | 3.4 (1.0) | 3.6 (1.1) | **0.031** |
| D-dimer, mean (SD), mmol/L | 1.7 (3.1) | 1.4 (3.1) | 1.9 (3.3) | 0.192 |
| **TOAST, n (%)** | | | | |
| LAA | 211 (81.5) | 83 (80.6) | 127 (80.9) | 0.951 |
| CE | 42 (16.2) | 17 (16.5) | 25 (16.0) | 0.918 |
| ODC | 3 (1.2) | 3 (2.9) | 0 (0) | 0.062 |
| UND | 4 (1.5) | 0 (0) | 4 (2.6) | 0.154 |
| **Infarction area, n (%)** | | | | |
| AC | 138 (53.1) | 58 (56.3) | 80 (51.0) | 0.397 |
| PC | 109 (41.9) | 38 (36.9) | 71 (45.2) | 0.183 |
| AC & PC | 12 (4.6) | 6 (5.8) | 6 (3.8) | 0.652 |
| **EVT** | | | | |
| OPT, median (P25, P75), min | 444 (317, 667) | 423 (293, 640) | 480 (353.5, 676.5) | 0.108 |
| ORT, median (P25, P75), min | 526 (400, 735) | 498 (360, 685) | 560 (428, 775) | **0.015** |
| General anesthesia, n (%) | 89 (34.2) | 27 (26.2) | 63 (40.1) | **0.021** |
| Stent retriever, n (%) | 165 (55.4) | 59 (57.3) | 106 (67.5) | 0.094 |
| Aspiration, n (%) | 186 (71.5) | 73 (70.9) | 113 (72.0) | 0.847 |
| Stent implantation, n (%) | 103 (39.6) | 41 (39.8) | 62 (39.5) | 0.719 |
| Balloon dilatation, n (%) | 119 (45.8) | 42 (40.8) | 77 (49.0) | 0.191 |
| Artery thrombolysis, n (%) | 14 (5.4) | 4 (3.9) | 10 (6.4) | 0.385 |
| NOP, mean (SD), times | 0.93 (0.93) | 0.83 (0.97) | 1.01 (0.89) | 0.123 |
| mTICI ≥ 2b, n (%) | 243 (93.5) | 102 (99.0) | 141 (89.8) | **0.003** |
| Residual stenosis after recanalization, n (%) | 78 (30) | 28 (27.2) | 50 (31.8) | 0.422 |
| **Treatment of tirofiban** | | | | |
| EVT to Tirofiban, median (P25, P75), min | 0 (0, 49.5) | 0 (0, 33) | 0 (0, 52.5) | 0.431 |
| Onset to Tirofiban, median (P25, P75), min | 568 (428, 801) | 540 (388, 780) | 580 (453.5, 840) | 0.116 |
| Arterial injection, n (%) | 60 (23.1) | 22 (21.4) | 38 (24.2) | 0.594 |
| Duration of Tirofiban > 24h, n (%) | 79 (30.4) | 42 (40.8) | 37 (23.6) | **0.003** |
| **NIHSS at different time point, mean (SD), points** | | | | |
| After EVT | 16 (9, 23.75) | 10 (6, 18) | 18 (12, 29.5) | **< 0.001** |
| Before the treatment of tirofiban | 17.5 (12, 25) | 18 (10, 26) | 17 (13, 25) | 0.606 |
| At the end of tirofiban treatment | 15 (6, 22.75) | 6 (2, 13) | 19 (13, 30.5) | **< 0.001** |
| NIHSS at 72 hours | 14 (5.25, 23) | 5 (2, 15) | 19 (12, 30) | **< 0.001** |
| NIHSS at discharge | 11 (4, 19) | 4 (2, 10) | 15 (10, 29.5) | **< 0.001** |
| **Outcomes, n (%)** | | | | |
| Early neurological recovery at 72h | 76 (29.2) | 61 (59.2) | 15 (9.6) | **< 0.001** |
| Reocclusion within 72 hours | 25 (9.6) | 2 (1.9) | 23 (14.6) | **0.001** |
| sICH | 16 (6.2) | 1 (1.0) | 15 (9.6) | **0.005** |

BMI: body mass index; SBP: systolic blood pressure; DBP: diastolic blood pressure; NIHSS: National Institute of Health stroke scale; ASPECT: Alberta Stroke Program Early CT Score; NEUT: neutrophil; PLT: platelet; TG: triglyceride; T-Chol: Total-Cholesterol; LDL-C: low-density lipoprotein cholesterol; HCY: homocysteine; Fib: Fibrinogen; TOAST: Trial of Org 10 172 in acute stroke treatment; LAA: large-artery atherosclerosis; CE: Cardio-embolism; ODC: other determined cause; UND: undetermined cause; AC: anterior circulation; PC: posterior circulation; EVT: endovascular treatment; OPT: onset to puncture time; ORT: onset to reperfusion time; NOP: Number of passes; mTICI: modified Thrombolysis in Cerebral Infarction Score; sICH: symptomatic cerebral hemorrhage.

## Figure Legend

## SUPPLEMENTAL FIGURE 1. Factors affecting the effective response to tirofiban after EVT.
